# A bionic system with Fenton reaction and bacteria as a model for bioprocessing lignocellulosic biomass

**DOI:** 10.1186/s13068-018-1035-x

**Published:** 2018-02-08

**Authors:** Kejing Zhang, Mengying Si, Dan Liu, Shengnan Zhuo, Mingren Liu, Hui Liu, Xu Yan, Yan Shi

**Affiliations:** 1 School of Metallurgy and Environment, Central South University, Changsha, 410083 People’s Republic of China; 2 Chinese National Engineering Research Center for Control & Treatment of Heavy Metal Pollution, Changsha, 410083 People’s Republic of China

**Keywords:** Lignocellulose pretreatment, *Cupriavidus basilensis* B-8, Fenton reaction, Enzymatic hydrolysis

## Abstract

**Background:**

The recalcitrance of lignocellulosic biomass offers a series of challenges for biochemical processing into biofuels and bio-products. For the first time, we address these challenges with a biomimetic system via a mild yet rapid Fenton reaction and lignocellulose-degrading bacterial strain *Cupriavidus basilensis* B-8 (here after B-8) to pretreat the rice straw (RS) by mimicking the natural fungal invasion process. Here, we also elaborated the mechanism through conducting a systematic study of physicochemical changes before and after pretreatment.

**Results:**

After synergistic Fenton and B-8 pretreatment, the reducing sugar yield was increased by 15.6–56.6% over Fenton pretreatment alone and 2.7–5.2 times over untreated RS (98 mg g^−1^). Morphological analysis revealed that pretreatment changed the surface morphology of the RS, and the increase in roughness and hydrophilic sites enhanced lignocellulose bioavailability. Chemical components analyses showed that B-8 removed part of the lignin and hemicellulose which caused the cellulose content to increase. In addition, the important chemical modifications also occurred in lignin, 2D NMR analysis of the lignin in residues indicated that the Fenton pretreatment caused partial depolymerization of lignin mainly by cleaving the β-*O*-4 linkages and by demethoxylation to remove the syringyl (S) and guaiacyl (G) units. B-8 could depolymerize amount of the G units by cleaving the β-5 linkages that interconnect the lignin subunits.

**Conclusions:**

A biomimetic system with a biochemical Fenton reaction and lignocellulose-degrading bacteria was confirmed to be able for the pretreatment of RS to enhance enzymatic hydrolysis under mild conditions. The high digestibility was attributed to the destruction of the lignin structure, partial hydrolysis of the hemicellulose and partial surface oxidation of the cellulose. The mechanism of synergistic Fenton and B-8 pretreatment was also explored to understand the change in the RS and the bacterial effects on enzymatic hydrolysis. Furthermore, this biomimetic system offers new insights into the pretreatment of lignocellulosic biomass.

**Electronic supplementary material:**

The online version of this article (10.1186/s13068-018-1035-x) contains supplementary material, which is available to authorized users.

## Background

Most terrestrial biomass is sequestered in lignocellulose—the principal structural material in vascular plants. Accordingly, the biological decomposition of lignocellulosic materials plays an essential role in carbon cycling in terrestrial forested ecosystems [1, 2]. Simultaneously, lignocellulose is one of the richest renewable resources on the earth. Its biodegradation has been studied not only as an ecological process, but also in the context of both the development of environmentally friendly agricultural waste disposal processes and the production of “green” and renewable fuels and chemicals [3–5].

In nature, few organisms can metabolize lignocelluloses, because it has cellulose and hemicelluloses—components that can support microbial growth—embedded in lignin [6]. Lignin is a combinatorial and racemic polymer containing phenylpropanoid subunits connected via ether and carbon–carbon bonds [7]. These provide structural support and resistance for the plant cell wall against microbial attack and oxidative stress [8, 9]. Any organism that depends on lignocellulose as a source of carbon and energy must have some mechanisms to penetrate or disrupt this recalcitrant lignin barrier.

Thus far, studies on the biodegradation of lignocellulose have mainly focused on fungi. Wood decay basidiomycetes are the most efficient biodegraders of lignocellulose. They can break down and/or mineralize lignin with the help of two systems viz [10, 11]. However, the disadvantages of fungal pretreatment strategies are their low efficiency, considerable loss of carbohydrates and long residence times [12]. Although the microbial degradation of lignin has been most intensively studied in white-rot and brown-rot fungi, there are many reports of bacteria that are isolated from soil, wastewater, pulp, and black liquor that can decompose lignin [13, 14]. Compared to fungi, bacteria have a shorter growth cycle and stronger tolerance to harsh industrial conditions. However, bacteria generally only modify lignin or metabolize low molecular weight components of lignin [15]. Therefore, bacterial pretreatment to break lignin’s recalcitrance to lignocellulose is rarely reported.

As shown in nature, there are symbioses between bacteria and fungi for using lignocellulose. The fungi break the recalcitrant cell wall barrier and release the small molecular compounds that then become available for further degradation and assimilation by commensal bacteria [16, 17]. This interdependence of microbes is a key link in the turnover of nutrients and carbon in forest ecosystems. This symbiotic system provides a new idea for the use of lignin-degrading bacteria to treat natural lignocellulose. To shorten the long-term lignocellulose pretreatment by fungi, we considered replacing this pretreatment with new methods. Compared with white-rot fungi, which has a complete lignin-degrading enzyme system, brown-rot fungi harbor an oxidative radical-based system that causes non-enzymatic disruption of lignocellulose [18]. This oxidative step modifies the plant cell wall via the action of highly destructive oxygen-derived free radicals, such as the highly reactive hydroxyl radical (·OH), produced by extracellular Fenton chemistry (Fe^2+^ and H_2_O_2_). Hence, the biochemical mechanism of brown-rot fungi is an alternative to the energetic apparatus of lignocellulose breakdown employed by white-rot fungi. A few researchers have reported that the Fenton reaction induced by chemical reagents can reduce the molecular weight of lignin and enhance enzymatic saccharification of lignocellulosic biomass [19]. Moreover, Fenton reactions occur under mild conditions, and thus it is considered as an environmentally friendly process [20, 21].

In our previous work, *Cupriavidus basilensis* B-8 (here after B-8), isolated from the steeping fluid of eroding bamboo slips, was identified as a potential bacterium for lignin degradation [22]. Here, inspired by natural lignocellulose degradation systems, we hope to combine mild chemical pretreatment with B-8 to rapidly simulate fungal invasion of plant tissue. Herein, we used a low-cost Fenton catalyst (Fe^3+^ and H_2_O_2_) to pretreat the selected model of lignocellulosic biomass (rice straw, RS), and then combined it with B-8 to form a biomimetic system that then enhanced enzymatic hydrolysis. This biochemical model is of interest to lignocellulose biorefineries for the sustainable production of chemicals, materials and fuels from renewable plant resources, which are more cost-effective and energy efficient.

## Methods

### Microorganism and RS

The bacterial strain B-8 had been deposited in the China General Microbiological Culture Collection Center (CGMCC) with an accession number of CGMCC 4240. Pure B-8 was cultured aerobically on a rotary shaker at 30 °C with a speed of 125 rpm in Luria–Bertani broth medium for 18 h. The culture was used as seed culture for biological pretreatment. RS used in all the experiments was obtained from Jining (Shandong, China). RS was ground into powder, air-dried, and then sifted by 60-mesh first and then 100-mesh griddles to make sure the size of the obtained RS was between 150 and 250 μm. The processed RS was used as the feedstock for the pretreatment.

### Solution phase Fenton pretreatment

RS pretreatment by the Fenton catalyst was performed in an Erlenmeyer flask at room temperature with stirring at 120 rpm. The Fenton reagent (FeCl_3_, FeCl_2_·4H_2_O, and H_2_O_2_) was added to the RS suspension at a solid loading of 10% (w/v) to initiate the reaction. After incubation for 2 h, the pretreated RS slurry was washed with deionized water until the pH of the filtrate reached 6–7; it was then dried at 55 °C for 12 h for further analysis. The concentration and the ratio of the constituents of Fenton’s reagent (i.e., FeCl_2_, FeCl_3_, and H_2_O_2_) are key parameters in the Fenton reaction. FeCl_3_, FeCl_2_ and H_2_O_2_ were diluted to the desired concentrations that the ratio of Fe to H_2_O_2_ was 1:75 [21], and then set a series of concentration gradients on this basis. The optimal concentration of FeCl_2_ was investigated as the control group. Fenton-A is 0.04 M FeCl_3_ and 3 M H_2_O_2_, Fenton-B is 0.03 M FeCl_3_ and 2.25 M H_2_O_2_, Fenton-C is 0.02 M FeCl_3_ and 1.5 M H_2_O_2_, Fenton-D is 0.01 M FeCl_3_ and 0.75 M H_2_O_2_, and Fenton-E is 0.02 M FeCl_2_ and 1.5 M H_2_O_2_. All the chemicals were analytical grade and purchased from Sinopharm Chemical Reagent Co., Ltd., of China.

### Bacterial pretreatment with B-8

The biological pretreatment was carried out in a 500 mL Erlenmeyer flask with 2 g of RS and 200 mL of mineral salt medium. The composition of the mineral salt medium was as follows (g L^−1^ in de-ionized water): (NH_4_)_2_SO_4_, 2; K_2_HPO_4_, 1; MgSO_4_, 0.2; CaCl_2_, 0.1; FeSO_4_, 0.05; MnSO_4_, 0.02; KH_2_PO_4_, 1; pH 7.0. Here, 20 mL of the bacterial culture was centrifuged, and the collected cells were inoculated into the medium. The biological pretreatment used a gas bath shaker at 30 °C with a speed of 125 rpm. After 2 days of incubation, the bio-treated RS sample was collected and thoroughly washed with deionized water. It was then dried at 50 °C for further experiments.

### Enzymatic saccharification

Commercial cellulase Cellic (CTec2, Novozymes) with 12 filter paper units (FPU) g^−1^ biomass was used for enzymatic hydrolysis. The 0.5 g of RS were added to 20 mL of citrate buffer (50 mM, pH 4.8) supplemented with cycloheximide (20 μg mL^−1^) and tetracycline (20 μg mL^−1^) to prevent microbial contamination. 30 μL of cellulase was loaded into the mixture to start the reaction in a shaking incubator with the speed of 120 rpm at 50 °C for 72 h. After incubation, samples were collected and centrifuged for sugar analysis. The reducing sugar was measured via the DNS assay [23].

### Chemical composition measurement of RS

The chemical composition of the RS was determined according to the chemical analysis methods reported by Teramoto et al. [24]. Specifically, holocellulose content was determined via weighting the NaClO_2_-delignified residue; cellulose content was determined via weighting the insoluble residue of holocellulose in the 17.5% NaOH aqueous solution; lignin content was determined via weighting the insoluble residue in 72% sulfuric acid aqueous solution. All experiments were carried out in triplicate.

### Analytical methods

Scanning electron microscope (SEM): Samples obtained from various pretreatments coated with gold using a sputter coater were observed under a SEM (JSM-IT300LA, JEOL, Japan); atomic force microscopy (AFM): AFM was performed in tapping mode on a NanoManTM VS + MultiMode V scanning probe microscope (Veeco Company, USA); BET specific surface area test method (BET): The size distribution of RS was characterized using a Malvern Mastersizer 2000 particle size analyzer (Malvern Instruments, UK). X-ray diffraction (XRD): XRD method was used to detect the RS crystallinity index (CrI) using a TTR III X-ray diffractometer (Rigaku, Japan). The RS powders were laid on the glass sample holder and were analyzed under plateau conditions. The Ni-filtered Cu Kα radiation (*λ* = 0.154056 nm) was generated at 40 kV and a current of 18 mA. and the scan speed was 2° s^−1^ from 10° to 40° [25, 26]. CrI was calculated using the following equation:

$${\text{Crystallinity}} = \left[ {{{\left( {I_{002} - I_{\text{amp}} } \right)} \mathord{\left/ {\vphantom {{\left( {I_{002} - I_{\text{amp}} } \right)} {I_{002} }}} \right. \kern-0pt} {I_{002} }}} \right]$$

where *I*_002_ is the intensity of the (002) peak at about 2*θ* = 22.1^o^, and *I*_amp_ is the intensity of the background at about 2*θ* = 16.1^o^; Fourier transform infrared spectroscopy (FTIR): The background spectrum of pure potassium bromide was subtracted from that of the sample spectrum. The FTIR spectra of the samples were obtained with analysis performed on a 1760X Fourier transform infrared spectrometer (PerkinElmer, Shanghai, China) from 4000 to 500 cm^−1^; 2D-nuclear magnetic resonance (2D-NMR): Studies on lignin from RS were performed according to Wen et al. [27]. Lignin (40 mg) was dissolved in anhydrous pyridine (500 mL) and deuterated chloroform (1.6:1, v/v) along with *N* hydroxynaphthalimide (200 mL) as an internal standard (11.4 mg mL^−1^ in pyridine and deuterated chloroform) and chromium acetylacetonate (0.28 mg). Next, the phosphitylating reagent 2-chloro-1,3, 2-dioxaphospholane (100 mL) was added. The mixture was stirred for 10 min and subjected to Bruker (Billerica, MA) AVIII 500 MHz NMR spectrometer spectra.

## Results and discussion

### Effects of pretreatments on enzymatic hydrolysis

**Fig. 1:**
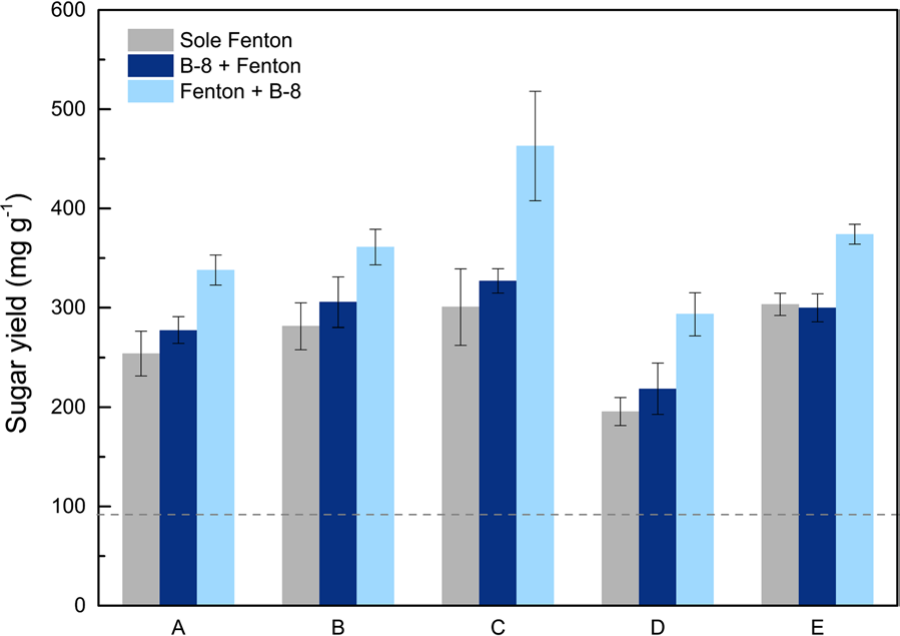
The effect of pretreatment conditions on the enzymatic hydrolysis of the pretreated RS. The sugar yield of untreated RS was pointed out with dashed line. The experimental groups Fenton-A is 0.04 M FeCl_3_ and 3 M H_2_O_2_, Fenton-B is 0.03 M FeCl_3_ and 2.25 M H_2_O_2_, Fenton-C is 0.02 M FeCl_3_ and 1.5 M H_2_O_2_, Fenton-D is 0.01 M FeCl_3_ and 0.75 M H_2_O_2_, and Fenton-E is 0.02 M FeCl_2_ and 1.5 M H_2_O_2_

To determine the feasibility of this bionic method, RS was treated with only the Fenton reaction, synergistic Fenton and B-8 pretreatment (Fenton + B-8), or synergistic B-8 and Fenton pretreatment (B-8 + Fenton). Herein, the digestibility of untreated and pretreated RS were presented in Fig. 1. During the Fenton pretreatment, H_2_O_2_ was first decomposed into hydroxyl or perhydroxyl radicals when the transition metal (Fe^3+^/Fe^2+^) presented. Then, the radicals randomly attacked the cell wall and caused lignocellulose structure breakdown. Generally, Fenton reaction was described as the following two reaction phases:

1 $${\text{Fe}}^{2 + } + {\text{H}}_{2} {\text{O}}_{2} \to {\text{Fe}}^{3 + } + {\text{OH}} + {\text{OH}}^{ - }$$

2 $${\text{Fe}}^{3 + } + {\text{H}}_{2} {\text{O}}_{2} \to {\text{Fe}}^{3 + } {-}{\text{OOH}}^{2 + } + {\text{H}}^{ + }$$

$${\text{Fe}}^{3 + } - {\text{OOH}}^{2 + } \to {\text{Fe}}^{2 + } + {\text{HO}}_{2} \cdot$$

$${\text{Fe}}^{2 + } + {\text{H}}_{2} {\text{O}}_{2} \to {\text{Fe}}^{3 + } + \cdot {\text{OH}} + {\text{OH}} ^{ - }$$

$${\text{Fe}}^{{ 2 { + }}} {\text{ + HO}}_{ 2} \cdot {\text{ + H}}^{ + } \to {\text{Fe}}^{{ 3 { + }}} { + } \cdot {\text{H}}_{ 2} {\text{O}}_{ 2}$$

$${\text{Fe}}^{{ 3 { + }}} {\text{ + HO}}_{ 2} \cdot \to {\text{Fe}}^{{ 2 { + }}} {\text{ + O}}_{ 2} {\text{ + H}}^{ + }$$

In the first phase, Fe^2+^ initiates and catalyzes the decomposition of H_2_O_2_ rapidly and results in the generation of ·OH. Meanwhile, Fe^3+^ gives rise to a complex chain reaction sequence. In contrast to the first reaction phase, the second phase is several orders of magnitude slower. Herein, we used the Fe^3+^ to reduce H_2_O_2_ into free ·OOH radicals under mild conditions. After pretreatment, the residues were subsequently submitted to enzymatic hydrolysis for 72 h. The RS pretreated with only H_2_O_2_ or only Fe(II)/Fe(III) was not effectively hydrolyzed by the cellulase (Additional file 1: Figure S1). In contract, the reducing sugar yields per gram of RS varied from 180.4 to 332.6 mg g^−1^, which were 1.8–3.4 times higher than that from untreated RS (~ 98 mg g^−1^, dashed line). This was primarily due to the destruction of the recalcitrant structure of the biomass, which improved the substrate hydrolyzability by enzymes. This indicated that Fenton pretreatment can imitate the rapid fungal invasion observed in natural symbiotic systems. More significantly, there was a universal improvement (15.6–56.6%) over Fenton pretreatment alone with Fenton + B-8 pretreatment. Higher digestibility (270.5–514.1 mg g^−1^) that was 2.7–5.2 times higher than untreated RS was obtained. In addition, the saccharification was weakened at higher concentrations of Fenton reagents. Similar results were obtained after B-8 pretreatment. This phenomenon was due to Fenton reaction that was too strong at higher concentrations which can severely damage the cellulose matrix [19]. The maximum production of sugars was obtained with 0.02 M FeCl_3_ and 1.5 M H_2_O_2_. The average yield of the reducing sugar increased 4.71-fold compared with untreated RS. This suggested that the synergistic B-8 and Fenton pretreatment greatly improve enzymatic hydrolysis under mild conditions; on the other hand, free ·OOH radicals produced by Fe^3+^ catalysis that were milder than the free ·OH radicals produced by Fe^2+^ catalysis were suitable for RS pretreatment. Table 1 summarizes the recent reports of Fenton reaction pretreatment and combined biological and chemical pretreatments. It showed that our improvements were quite competitive with methods described in the literature. Notably, the Fenton pretreatment was conducted at room temperature without external energy input, and the bacterial pretreatment required much less time than fungal pretreatments [28]. Accordingly, the Fenton + B-8 pretreatment was highly competitive over other biological pretreatments.

**Table 1:** Comparison of different pretreatments strategies and the increase of sugar yield as compared with the untreated substrate

Biomass	The pretreatment process	The increase of the sugar yield
Mixed hardwood [41]	Fenton (FeSO_4_ 0.1 M, H_2_O_2_ 2.5 M, 50 °C, 96 h) + hydrothermal treatment (190 °C, 10 min)	3.80-fold
Rice straw [21]	Fenton (0.05 M FeCl_3_, 2.5 M H_2_O_2_, 25 °C, 24 h)	3.85-fold
Cotton stalk [42]	*Phanerochaete chrysosporium* (fungal, 14 days, 39 °C)	< 1-fold
*Glycyrrhiza uralensis* [28]	H_2_SO_4_ (2.5%, 100 °C, 180 min) + *Phanerochaete chrysosporium* (fungal, 21 days, 28 °C)	2-fold
Rice straw [43]	NaOH/urea (4%/6%, − 10 °C, 240 min) + *Sphingobacterium* sp. LD-1 (bacterial, 4 days, 30 °C)	5.4-fold
Rice straw (in this study)	Fenton (0.02 M FeCl_3_, 1.5 M H_2_O_2_, 25 °C, 120 min) + *Cupriavidus basilensis* B-8 (bacterial, 2 days, 30 °C)	4.71-fold

The results of synergistic B-8 and Fenton pretreatment had a yield of reducing sugar that was far lower than the Fenton + B-8 pretreatment. This indicated that the effect of sole B-8 treatment was very weak, and it was difficult for B-8 to break untreated RS. The very positive effect on the reducing sugar yield by Fenton + B-8 pretreatment showed that the Fenton reaction changed the biomass structure and modified lignin. This facilitated lignin residues that were partly utilized and/or further modified by B-8. Lignin removed by bacteria after Fenton pretreatment further exposed more hemicellulose and cellulose and improved the hydrolysis performance. Fenton pretreatment created conditions for bacterial treatment, validated the feasibility of the model for bioprocessing, and allowed the lignin-degrading bacteria to proceed after chemical pretreatment. Obviously, this bionic method was very different from the real symbiosis system and evolves special mechanism. Next, to explore the mechanism, we carried out a series of detailed characterization steps.

### Effects of pretreatments on chemical components

**Fig. 2:**
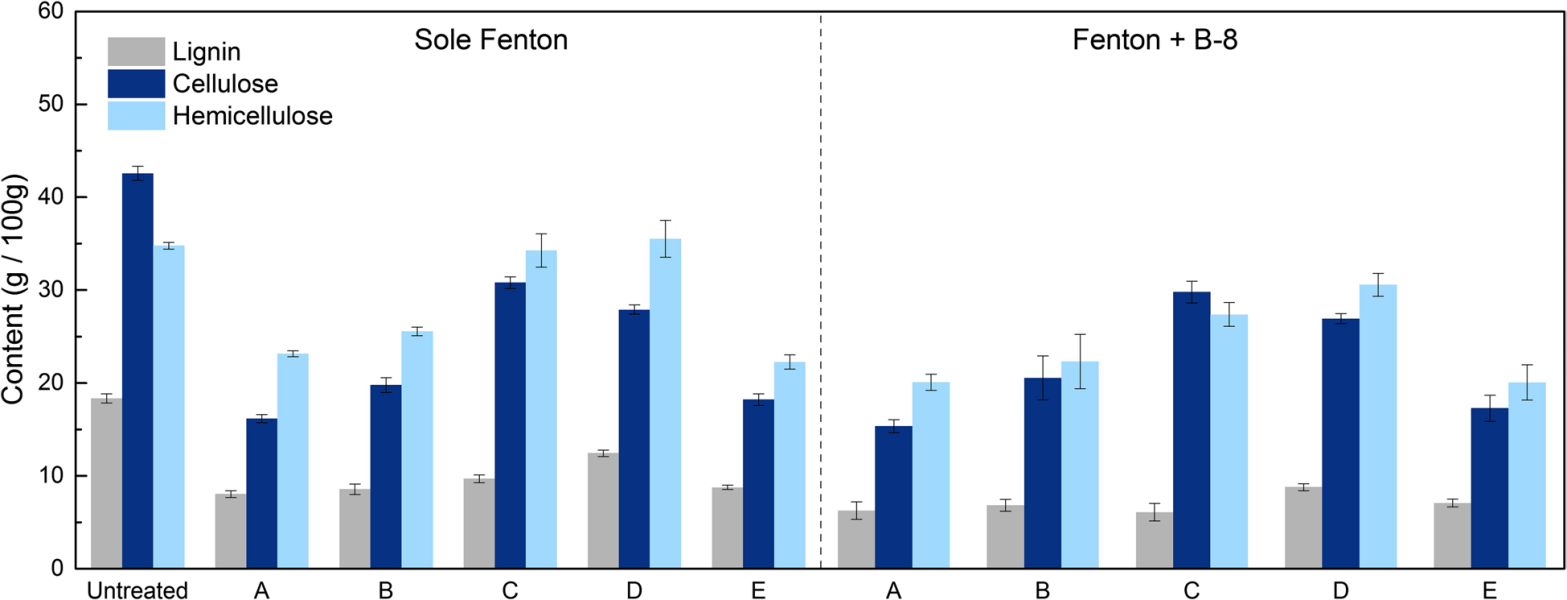
The effect of pretreatment conditions on the chemical composition of the pretreated RS. The experimental groups Fenton-A is 0.04 M FeCl_3_ and 3 M H_2_O_2_, Fenton-B is 0.03 M FeCl_3_ and 2.25 M H_2_O_2_, Fenton-C is 0.02 M FeCl_3_ and 1.5 M H_2_O_2_, Fenton-D is 0.01 M FeCl_3_ and 0.75 M H_2_O_2_, and Fenton-E is 0.02 M FeCl_2_ and 1.5 M H_2_O_2_

The untreated RS was found to mainly contain 42 g of cellulose, 35 g of hemicellulose and 18 g of lignin in 100 g of initial total biomass. The composition of the different chemical components in the residues after various pretreatment steps was seen in Fig. 2. A compositional analysis of the RS after pretreatment using the Fenton’s reagent for 2 h revealed that the amounts of lignin were significantly reduced to 7.8–12.7 g per 100 g of initial total RS with the removal rate of 29.4–56.6%, this was probably due to the oxidation of the lignin. The content of the cellulose obviously decreased to 15.8–31.2 g per 100 g. The Fenton reaction was fast and harsh, and hydrogen peroxide radicals non-selectively attacked the cell walls, results in rapid degradation of the cellulosic substrates. Combined with the results of the previous enzymatic hydrolysis analysis (“Effects of pretreatments on enzymatic hydrolysis” section), Fenton pretreatment decreased the content of cellulose, but generally improved sugar yields. This might be because free radicals produced by the Fenton reaction act on cellulose, resulting in oxidation and the creation of more sites for the cellulases to bind, improving the hydrolysis. However, fiber surface fibrillation generally occurs under mild reaction conditions, pretreatments with high concentrations of Fenton’s reagents can cause severe damage to the cellulosic fibers [29]. This also explained why Fenton-C could retain more cellulose and had a better effect on the enzymatic hydrolysis than Fenton-A and Fenton-B with high concentrations of Fenton reagents. Furthermore, relatively high amounts of hemicellulose loss (26–38%) were also observed under severe pretreatment conditions (Fenton-A, Fenton-B and Fenton-E).

To investigate the effect of Fenton + B-8 pretreatment on the content of major components, RS pretreated with the Fenton reagent was further treated by B-8. As shown in Fig. 2, after B-8 pretreatment, the contents of cellulose, lignin and hemicellulose were adjusted to 14.8–30.6, 5.4–9.0 and 18.7–31.4 g in 100 g of initial total RS, respectively. The results showed that B-8 removed part of the lignin and hemicelluloses while retaining the cellulose. These effects of B-8 were crucial to obtain high levels of enzymatic digestibilities in the Fenton + B-8 pretreatment system. These results illustrated that B-8 impacted the pretreatment of the lignocellulosic biomass. Physical and chemical characterization was next performed under optimal conditions (Fenton-C) to elucidate the role of B-8 in the pretreatment.

### Surface morphology change of RS

**Fig. 3:**
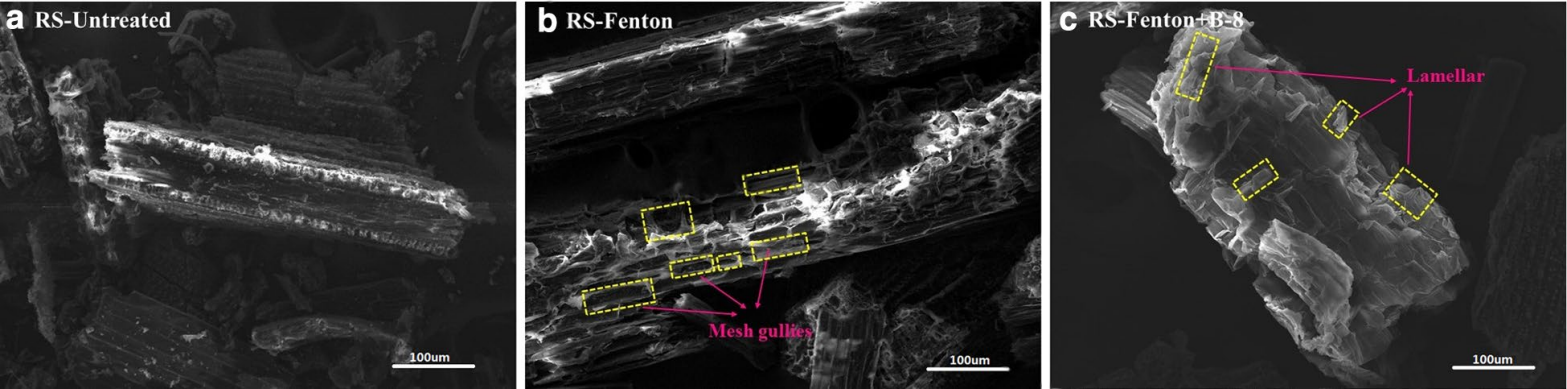
SEM images of the untreated and pretreated RS. **a** The untreated RS had a regular and intact morphology. **b** After Fenton pretreatment, the compact surface of RS was completely broken and formed a lot of mesh gullies. **c** After B-8 pretreatment, RS appeared a loose lamellar structure

Efficient enzymatic hydrolysis depends on the availability of the cellulose system to cellulase. The removal of lignin and hemicellulose could enhance hydrolyzability, but cellulase usually acts on the surface of solid cellulose [30]. Surface morphology visually showed changes in the solid surface structure as well as the morphological structures of untreated RS, Fenton-RS and Fenton + B-8-RS fractions as captured by SEM in Fig. 3. The untreated RS has a regular and intact morphology with a smooth and compact surface. When exposed to the Fenton reagents, the smooth surface of the RS was completely broken and formed a lot of mesh gullies. The partially separated fiber and obviously collapsed framework presented loose RS. This suggested that the Fenton reactions caused rapid depolymerization on the surface, but the cellulose structure was not exposed. After B-8 pretreatment, the mesh portion on the surface of the RS became smooth. The hemicellulose and lignin were partially removed, leaving a loose lamellar structure. The raised portion of the surface mesh formed by the Fenton reaction might became the “landing point” of B-8. Thus, B-8 further modified the RS surface.

**Fig. 4:**
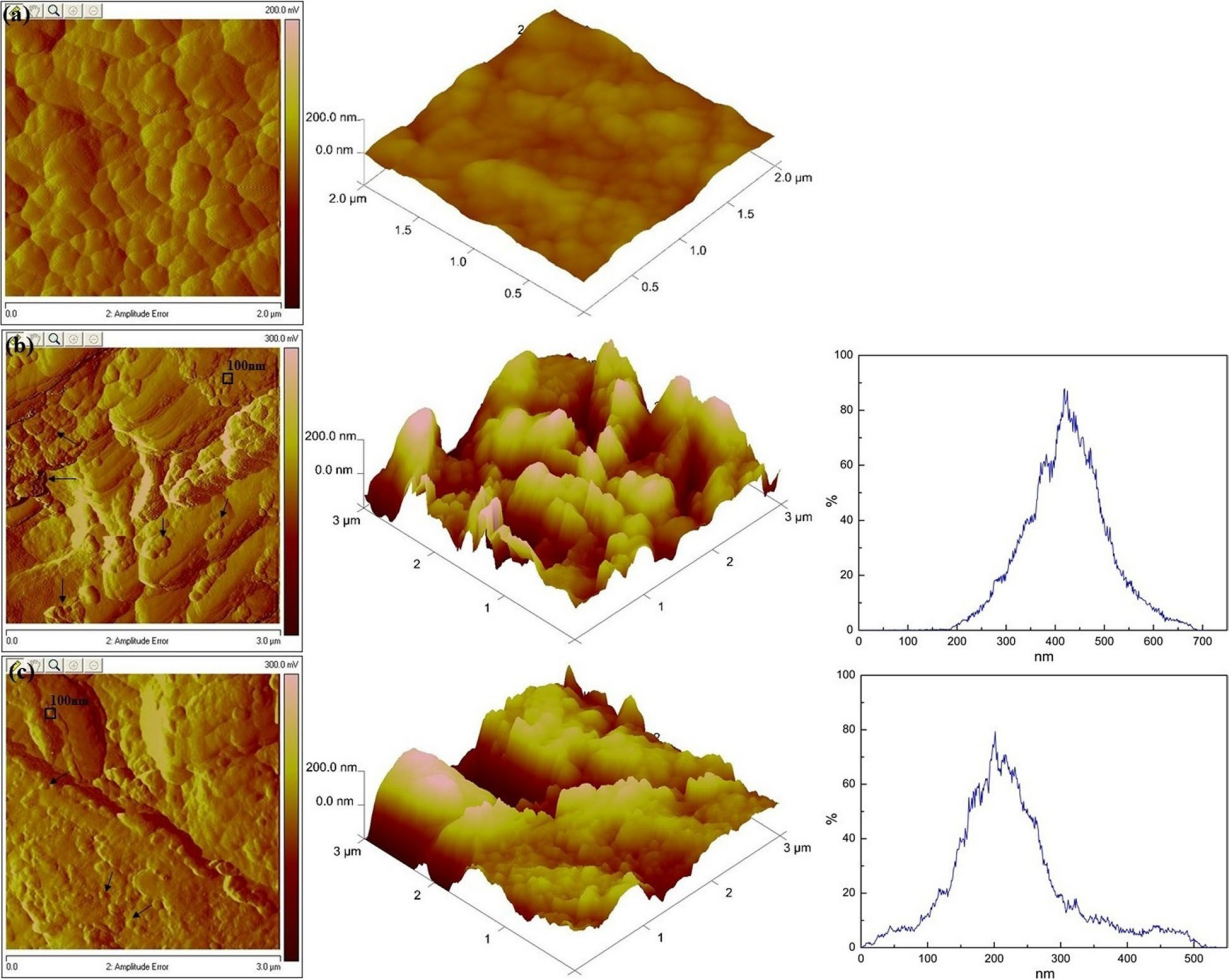
AFM amplitude images,3D images and height distribution images (from left to right): **a** the untreated RS presented a smooth surface; **b** RS-Fenton surface became noticeably rough with the appearance of highlighted hydrophilic hemicellulose sites and hydrophobic lignin droplets (20–100 nm, pointed out with the arrow); **c** RS-Fenton + B-8, the globular shapes of the deposited lignin decreased

Nanometer-scale AFM was used to image RS to observe more details of the surface changes after pretreatment in Fig. 4. In the 3D image, the untreated RS presented a smooth surface, and the surface morphology of RS became noticeably rough after pretreatment. For quantitative analysis, the roughness average (*R*_a_) and the maximum vertical distance between the highest and lowest data points (*R*_max_) in the AFM image are listed in Table 2. All the roughness parameters increased significantly due to pretreatment with Fenton or Fenton + B-8.

**Table 2:** Physiochemical characteristics of untreated and pretreated RS

RS	Crystallinity^a^	*R*_a_ (nm)^b^	*R*_max_ (nm)^b^	SSA (m^2^ g^−1^)^c^	PV (cm^3^ g^−1^)^c^
Untreated	0.489	12.4	111	0.647	0.0021
Fenton	0.575	64.2	692	0.936	0.0023
Fenton + B-8	0.644	64.6	523	0.967	0.0033

^a^Crystallinity calculated from XRD analysis. ^b^ *R*_a_: average surface area roughness; *R*_max_: the maximum vertical distance between the highest and lowest data points in the AFM image. ^c^ SSA: specific surface area; PV is the total pore volume calculated from BET analysis

Generally, in the AFM amplitude images, the tip adhered more strongly to the hydrophilic area, which appeared lighter in color [31]. Untreated RS surfaces were predominantly hydrophobic (indicated by darker phase images). This was the main reason for its low digestibility. Images taken after Fenton pretreatment of the outer cell wall phase showed several lighter patches that corresponded to regions abundant in hydrophilic species. This increased the availability of the enzymes. Correspondingly, the *R*_a_ and *R*_max_ of the Fenton-RS were 5.17 and 6.23 times higher than untreated RS, respectively. A thin layer of deposits (likely hemicellulosic polymers) covered the surface. Moreover, the hydrophobic lignin droplets observed after acidic pretreatments were also seen on RS surfaces [32]. This was attributed to lignin migration to the outer surface, and the surface lignin droplets were easily removed due to the reduced binding to carbohydrate polymers. In fact, the color of the treated RS became dark brown, which confirmed this point. After Fenton + B-8 pretreatment, the globular shapes of the deposited lignin decreased, possibly because of the effects of B-8 on lignin droplets. The modified lignin was depolymerized to lower lignin contents. Some of this was utilized by B-8. The roughness parameters of RS-Fenton + B-8 were still much higher than those of the RS-control, and the *R*_a_ of RS-Fenton + B-8 was slightly above the RS-Fenton. In addition, the reduction of *R*_max_ was due to the role of B-8, which changed the height distribution of the RS surface. There was a clear movement of the height distribution peak toward lower classes, just as the B-8 trimmed the raised surface.

The variations in the SSA and PV were identified by N_2_ sorption at 77 K. Table 2 showed that both the SSA and PV of the pretreated RS increased obviously. For Fenton pretreatment, the increase in SSA due to the destruction of the lignocellulose structure by the free radical provided a greater surface area for subsequent bacterial treatment. After B-8 pretreatment, the increase in SSA and PV resulted from the depolymerization of lignin droplets and solubilization of the hemicellulose. The higher SSA and PV gave enhanced interfacial interactions between RS and the enzymes. This led to improved digestibility.

**Fig. 5:**
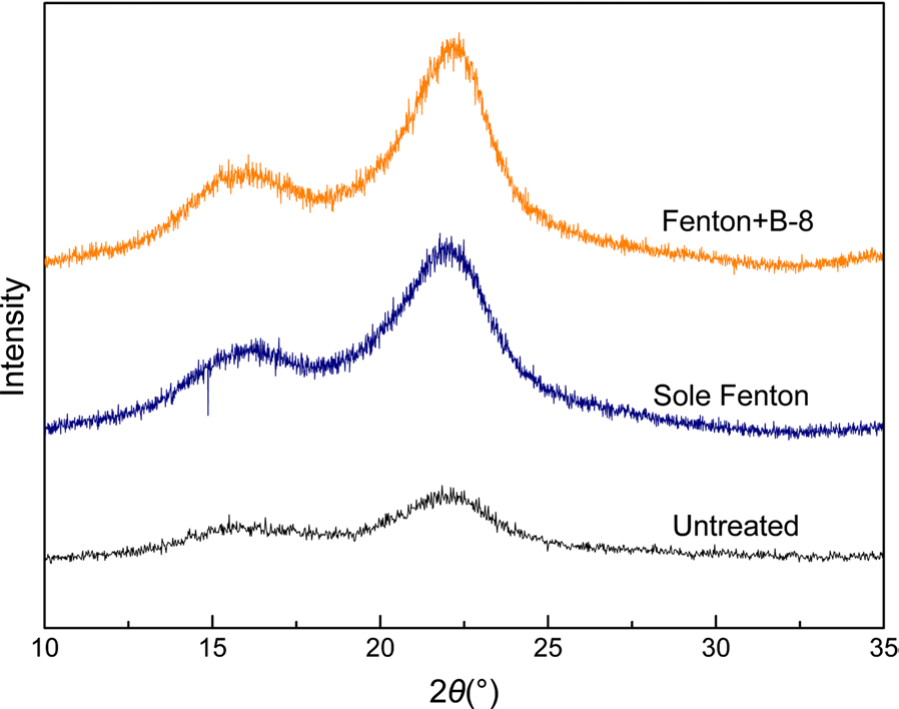
XRD patterns of the untreated and pretreated RS samples: the main diffraction peaks of 16.1° and 22.1° were assigned to the crystalline structures of cellulose I (101) and (002), respectively

The XRD assay was a reliable method to evaluate RS structural transformation. XRD patterns of untreated and pretreated RS were presented in Fig. 5. The main diffraction peaks of 16.1° and 22.1° were assigned to the crystalline structures of cellulose I (101) and (002), respectively [33]. The calculated crystallinity index (CrI) values were 0.489, 0.575 and 0.644 for untreated RS, Fenton-RS and Fenton + B-8-RS, respectively. After the Fenton reaction, the crystallinity increased by 17.6%, which suggested that pretreatments removed some amounts of the non-crystalline components. The RS structure that composed the crystalline regions was recalcitrant and was not easily destroyed by the Fenton reagent. Lignin migration to the outer surface exposed the internal cellulose surfaces, which increased the proportion of crystalline cellulose. After B-8 treatment, the crystallinity slightly increased to 0.644. The increase in cellulose CrI was due to the amorphous hemicellulose and lignin was partially removed. The lignin and crosslinked hemicellulose hindered hydrolysis in RS were stepwise destroyed by the Fenton + B-8 pretreatment.

Based on these results, we hypothesized that the Fenton pretreatment destroyed the recalcitrant structure of RS that was difficult to be bio-activated. The increased surface roughness formed many hydrophilic sites that facilitated cell adhesion and growth. The B-8 then rapidly attached to the hemicellulose hydrophilic sites and acted on the lignin droplets exposed on the surface. The removed lignin and hemicellulose content further enhanced the hydrolysis.

### Changes in chemical structure caused by pretreatment

**Fig. 6:**
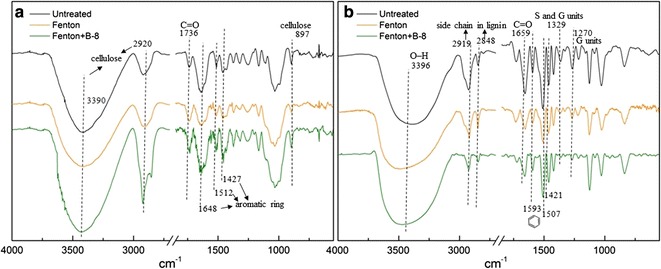
FTIR spectra of untreated and pretreated RS and lignin samples. **a** FTIR spectra of RS samples. The broad bands in the 3400–3300 cm^−1^ region, the band at 2920 and 897 cm^−1^ were cellulose characteristic peaks. The shoulder peak at 1736 cm^−1^ which associated with the carbonyl band (C=O) in lignin and/or hemicelluloses. The peaks at 1648, 1512 and 1427 cm^−1^ representing the lignin aromatic ring structure. **b** FTIR spectra of isolated lignin from RS samples. The shoulder peak at 3396 cm^−1^ derived from the O–H stretching. The absorption peaks at 2919 and 2848 cm^−1^ were the structure of the side chain in lignin. The peak at 1659 cm^−1^ derived from conjugated carbonyl (C=O). The characteristic peaks of the benzene ring skeleton appeared at 1593, 1507 and 1421 cm^−1^. The absorption peaks of S units and condensed G units at 1329 cm^−1^. G units C=O stretch at 1270 cm^−1^

The FTIR spectra of untreated RS, Fenton-RS and Fenton + B-8-RS were presented in Fig. 6a. The broad bands in the 3400–3300 cm^−1^ region were O–H stretching vibrations of cellulose, and the band at 2920 cm^−1^ was attributed to the C–H stretching within the methylene of cellulose [34]. The OH bending of adsorbed water was at 1639 cm^−1^, and the typical of β-glycosidic bonds in cellulose was at 897 cm^−1^ [35]. After Fenton pretreatment, the intensity of cellulose characteristic peaks significantly decreased. This indicated that the structure of cellulose was destroyed by free radicals. However, the intensity of these cellulose bands increased after B-8 pretreatment, revealing that cellulose was well-preserved in B-8 pretreatment. The intensity of the shoulder peak at 1736 cm^−1^ which associated with the carbonyl band (C=O) in lignin and/or hemicelluloses had been increased, corresponding to an increase in hemicellulose content by Fenton pretreatment. In addition, the intensity of the peaks representing the lignin aromatic ring structure also showed changes, including aromatic ring C=C stretching at 1648 cm^−1^, C–O stretching at 1512 cm^−1^, and aromatic skeletal vibrations at 1427 cm^−1^. After Fenton pretreatment, the intensity of these lignin characteristic peaks decreased, indicating that lignin was partly destroyed and removed. However, these peaks were enhanced after B-8 pretreatment, indicating that B-8 depolymerized lignin into small aromatic ring structures.

To further study the effects of B-8 on lignin, we extracted the lignin from RS. The FTIR spectra of lignin separated from untreated RS, Fenton-RS and Fenton + B-8-RS were presented in Fig. 6b. The spectra of lignin were similar before and after treatment only the absorption peak intensity was slightly different. This suggested that the overall structure of lignin was not destroyed to a large extent by chemical and bacterial pretreatment, only some chemical bonds were influenced. After Fenton pretreatment, the intensity of the shoulder peak at 3396 cm^−1^ derived from the O–H stretching increased, which might be attributed to the role of demethoxylation. The absorption peaks at 2919 and 2848 cm^−1^ were from C–H asymmetric and symmetrical stretching vibration of CH_3_ and CH_2_, respectively. This implied that the structure of the side chain in lignin was changed. The peak at 1659 cm^−1^ in the carbonyl region was derived from the absorption of the conjugated carbonyl (C=O). The characteristic peaks of the benzene ring skeleton appeared at 1593, 1507 and 1421 cm^−1^, and the decrease in those absorption peaks intensity indicated that the benzene ring structure of lignin was attacked by free radicals. In addition, both the absorption peaks of typical S units and/or condensed G units at 1329 cm^−1^ and C=O stretch of G units at 1270 cm^−1^ also decreased. These changes of functional groups further proved that decomposition of the stable chemical structures of the residual lignin was extensive. After B-8 pretreatment, the hydroxyl structure, side chain structural unit, conjugated carbonyl and G units were all relatively reduced. These changes indicated that the modified lignin was depolymerized by B-8.

**Fig. 7:**
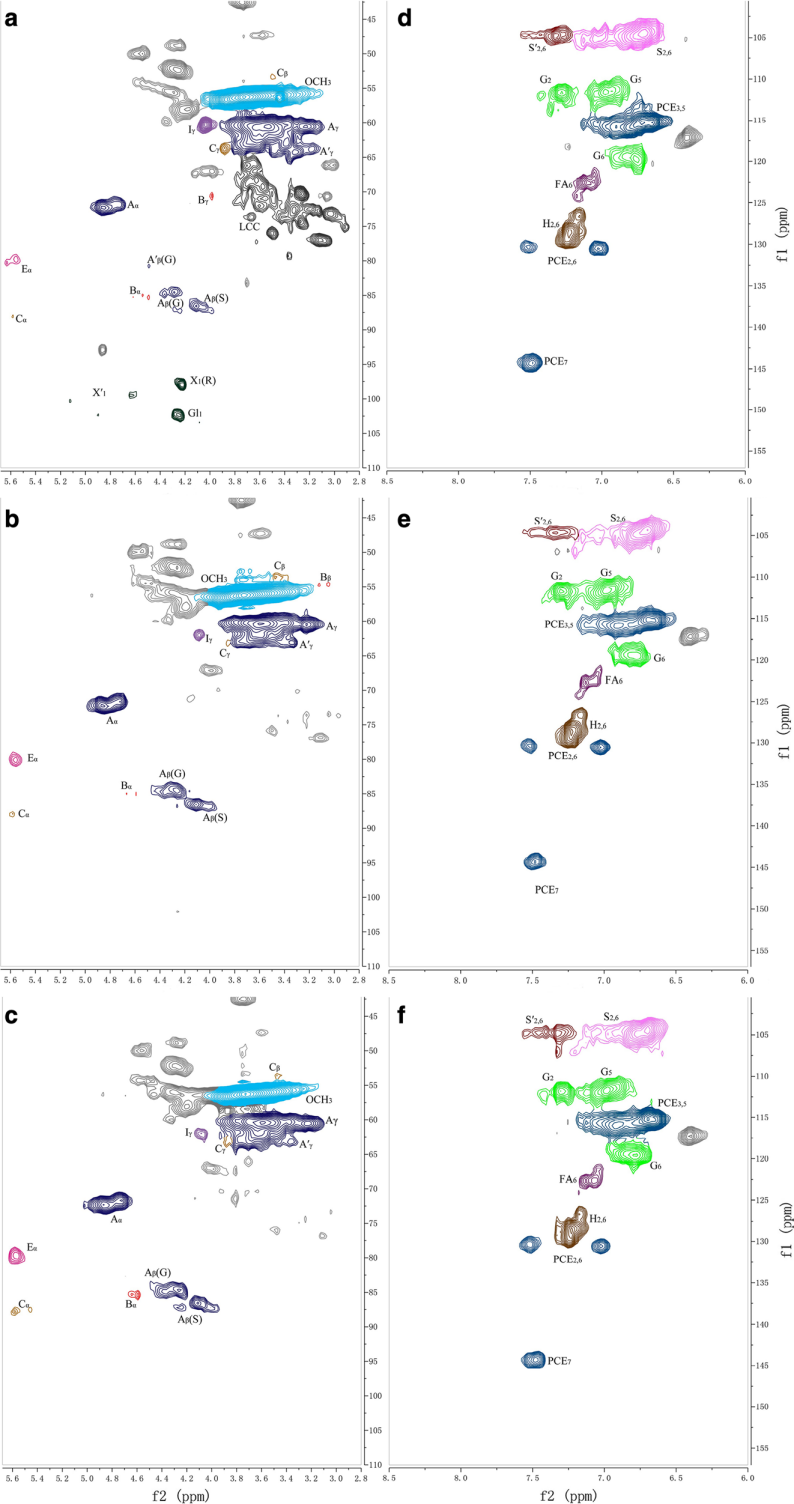
Two-dimensional (2D) NMR spectra (HSQC experiments) at the gel state of: **a**/**d** the lignin of RS-Control; **b**/**e** the lignin of RS-Fenton; and **c**/**f** the lignin of RS-Fenton + B-8

**Fig. 8:**
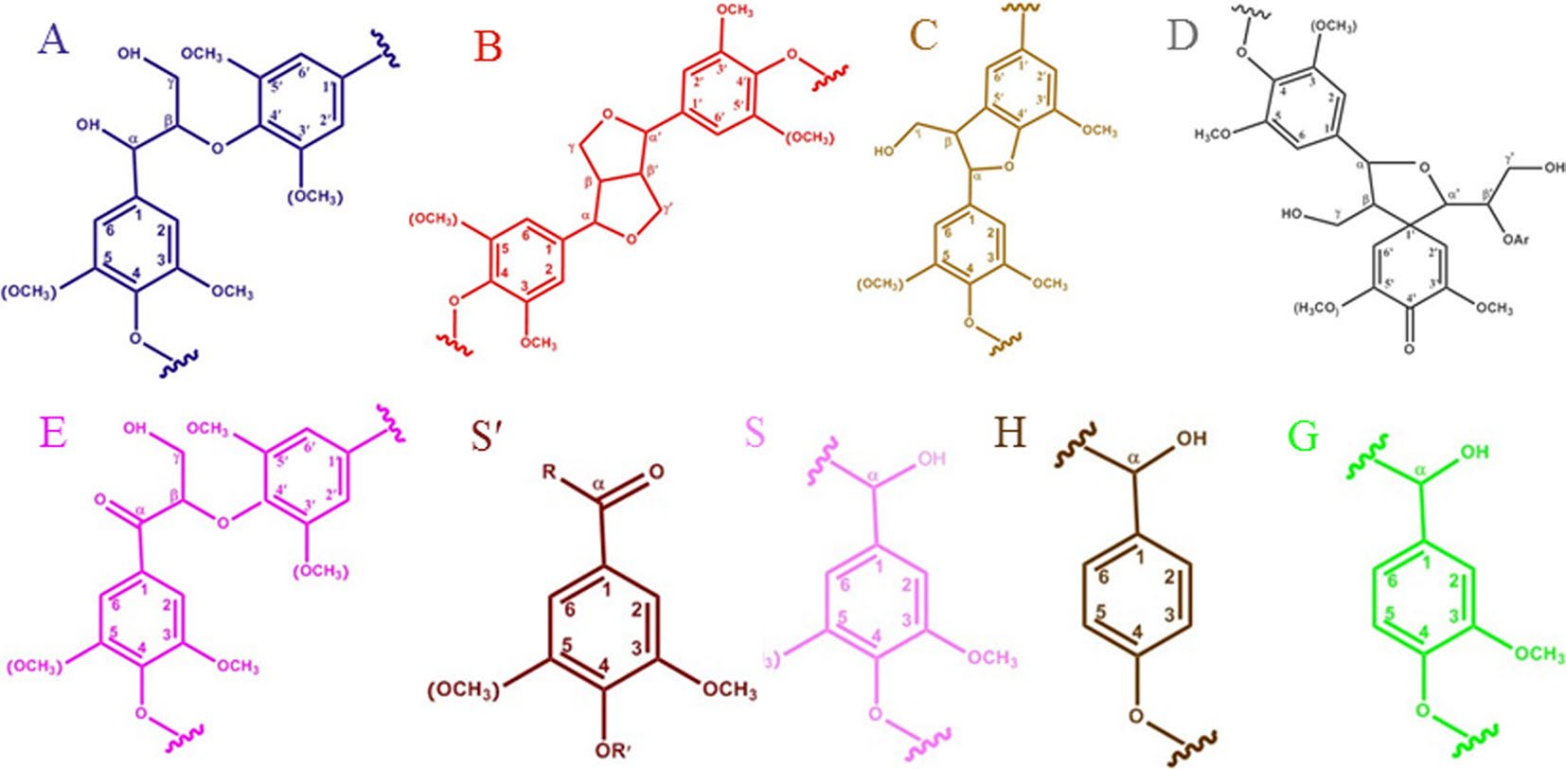
Two-dimensional (2D) NMR spectra (HSQC experiments) at the gel state of: the lignin structures identified are shown: (A) β-*O*-4′ substructure; (B) resinol substructure; (C) phenylcoumaran substructure;  (D) spirodienone substructure; (G) guaiacyl unit; (S) syringyl unit; (S′) C_α_-oxidized S unit; (E) *α,* β-diaryl ether substructures; (H) para-hydroxy-phenyl unit (R, lignin or OH; R′, H or lignin)

To further elucidate the lignin structure, two-dimensional hydrocarbon correlation spectra (2D-HSQC) analysis was carried out for characterization of the lignin from untreated and treated RS. The expanded spectra of the side chain and aromatic regions were shown in Figs. 7, 8. The data indicated that the overall structure of lignin was not drastically modified by the Fenton pretreatment, all the typical cross-signals of lignin remained visible. These included the well-resolved signals of β-*O*-4′ (A), β-β′ (resinol-type, B) and β-5′ (C) side-chain linkages together with strong aromatic signals of syringyl (S), guaiacyl (G) and *p*-hydroxyphenyl (H) lignin units. However, the relative intensities of some chemical bonds were apparently affected. Minor spirodienones (D) were not detected. The relative abundance of the main inter-unit linkages (given as per aromatic units and as percentage of total side-chains involved) were calculated based on the HSQC spectra (Additional file 2: Table S1). Abundances of lignin side-chain linkages and ratio of H, G and S were presented in Table 3.

**Table 3:** Semi-quantitative of the lignin polymer in the untreated and pretreated RS

	Untreated	Sole-Fenton	Fenton + B-8
β-*O*-4′ aryl ethers (A)	81.3	76.2	71.6
β-β′ resinols (B)	10.8	17.2	14.9
β-5′ phenylcoumarans (C)	7.9	6.6	13.5
β-1′ spirodienones (D)	0	0	0
Lignin aromatic unit^a^			
H (%)	6.1	7.8	9.3
S (%)	64.3	59.7	59.9
G (%)	29.6	32.5	30.8
S/G ratio	2.17	1.83	1.94

^a^Molar percentages (H + G + S = 100)

After Fenton pretreatment, the data indicated that the relative abundances of the β-*O*-4′ substructures were significantly reduced (from 81.3 to 76.2% of the total side-chains), and free radicals also modified that of the β-β′ substructures (increased by 6.4% of total side-chains). The relative abundances of β-5′ substructures decreased slightly (from 7.9% to 6.6% of total side-chains), the signal of C_γ_–H_γ_ in β-β′ (resinol) (B) almost disappeared, and a new signal for C_β_–H_β_ in β-β′ was found. This indicated that the β-*O*-4′ substructures were broken, and lignin was partially depolymerized by the Fenton reaction. As mentioned above, the Fenton reaction removed 36% of the lignin, meaning that the Fenton pretreatment destroyed a large amount of β-*O*-4′ substructures. However, a small increase in the oxygenated moieties was seen in the HSQC spectrum via the small signal corresponding to aliphatic C_α_–H_α_ correlation in C_α_-oxidized β-*O*-4′ substructures (E). This revealed that some oxidative alterations in the lignin structure occurred. The methoxyl peak at δC/δH 54–57/3.1–4.5 ppm also decreased, indicating that the radicals attacked lignin aromatic rings to cause demethylation, which was consistent with other studies [19]. The polysaccharide signals and possible lignin–carbohydrate linkages LCCs (δC/δH 65.7–79.3/2.8–3.7 ppm) were disappeared, demonstrating that carbohydrates in the RS lignin were almost depolymerized. And, lignin’s close association with hemicellulose was disrupted by the Fenton reagents. In the aromatic region, the change in S/G ratio is an important indicator for characterizing lignin structure change during pretreatment (Table 3). The reduction in the S/G ratio (2.17–1.83) implies that the Fenton pretreated lignin had a smaller molecular weight. The aromatic C_2_–H_2_ and C_6_–H_6_ correlation signal in C_α_-oxidized S units (S′) was also comparatively lower. The signal strength of the S and G units were reduced, perhaps because the S unit or G unit had been demethylated to form a common condensed H-type unit. Moreover, the increased H units increased the accessibility of hydrolytic agents, as the H units were essentially all terminal [36].

After B-8 treatment, the relative abundance of the β-*O*-4′ substructures decreased slightly (from 76.2 to 71.6% of total side-chains). The reduction also occurred on the β-β′ substructures. The signal of C_β_–H_β_ in β-β′ (resinol) (B) disappeared, accompanied by a significant increase in the structure of β-5′ (from 6.6 to 13.5% of total side-chains). The signals of B_α_ and C_α_ showed comparatively higher intensity. This implies that B-8 mainly broke down the lignin formed by β-*O*-4′ substructures. The β-*O*-4′ substructures were mainly composed of S units, and the broken β-*O*-4′ substructures might be transformed into *β*-5 substructures. The peaks of C_2_–H_2_ and C_6_–H_6_ in oxidized S units (S′) increased in the aromatic region; other signals did not change significantly. Besides, the G-type lignin unit linked by β-aryl ether was more easily depolymerized and dissolved in the medium than the S-type by B-8 pretreatment process. Thus, the ratio of S/G increased slightly. In previous research, we confirmed that the β-ketoadipate central pathway for ferulate degradation existed in B-8 by genomic analysis [37]. Ferulate served as the model compound for G units. In conclusion, the Fenton pretreatment caused partial depolymerization of lignin mainly by cleaving the β-*O*-4 linkage and by demethoxylation to remove the S and G units. B-8 could depolymerize amount of the G units by cleaving the β-5 linkages that interconnect the lignin subunits.

### Summary of the mechanism of Fenton + B-8 pretreatment

**Fig. 9:**
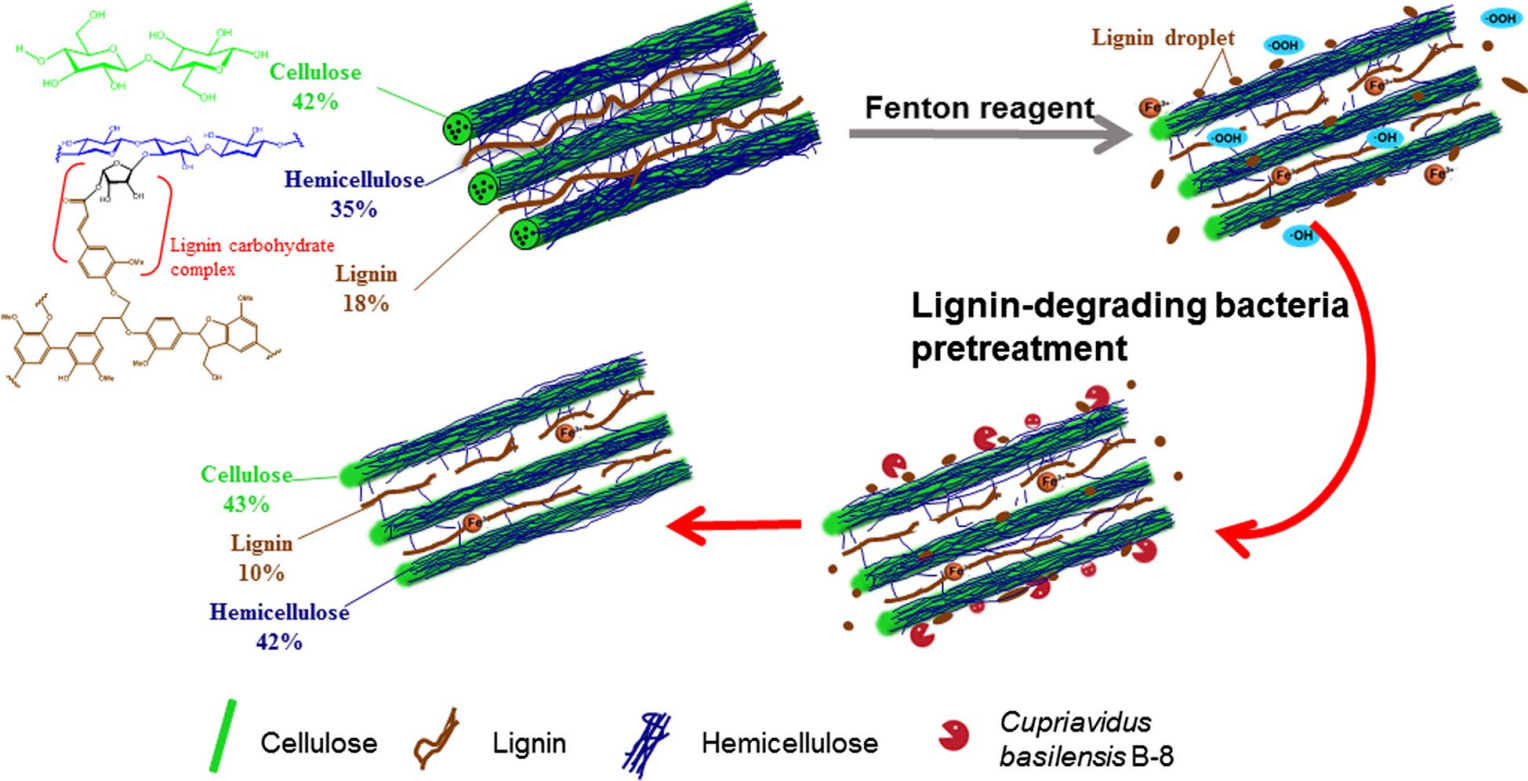
Mechanism diagram of Fenton + B-8 pretreatment: Fenton pretreatment changed the surface morphology of the RS. Free radicals cleaved the internal glycosidic bonds of cellulose and hemicellulose, caused fiber surface oxidation, and disrupted lignin’s close association with hemicellulose. Simultaneously, the lignin droplets migrated to the surface. When bacterial cells were inoculated into this system, they adhered to the surface of the RS to further cleave and/or dissolve the lignin as modified by the Fenton pretreatment

In summary, these analyses supported the hypothetical biomimetic mechanism which is depicted in Fig. 9. Fenton pretreatment simulated rapid fungal erosion of biomass, and changed the surface morphology of the RS, such that the increase in roughness and hydrophilic sites enhanced lignocellulose bioavailability. Free radicals cleaved the internal glycosidic bonds of cellulose and hemicellulose, caused fiber surface oxidation, and disrupted lignin’s close association with hemicellulose. Simultaneously, the lignin droplets migrated to the surface; lignin inter-unit linkages were cleaved by Fenton pretreatment which was conducive to further depolymerization of lignin by bacteria. In addition, lignin can absorb iron to form lignin–metal complexes through spontaneous chelation [19]. The lignin–metal complex may act as a capping agent to inhibit lignin re-condensation [19]. When bacterial cells were inoculated into this system, they adhered to the surface of the RS. Bacteria harbor versatile pathways for metabolizing complex substances, including recalcitrant aromatic substances ranging from simple phenols to the highly complex lignin polymer. Laccase, lignin peroxidase, and manganese peroxidase are the most well-studied enzymes involved in lignin degradation [38]. In our previous work, gene sequence and enzyme activity of a laccase has been confirmed in B-8 [37]. Laccase can degrade and/or dissolve the lignin to form a mixture of oxidative degradation products [37]. The laccase system is reported preferentially to attack phenolic substrates, and it can self-generate radicals by releasing the phenolic compounds from lignin to create a sustainable redox environment to achieve lignin depolymerization and modification [39]. The demethoxylation of Fenton reaction formed more phenolic hydroxyl groups. Consequently, the B-8 used a laccase system to further cleave and/or dissolve the lignin as modified by the Fenton pretreatment. Generally, lignin with higher guaiacyl content absorbs more cellulase [40]. In particular, B-8 could depolymerize amount of the G units as shown by the 2D NMR analysis and genomic analysis [37]. In addition, the B-8 also had a positive effect on the removal of the hemicellulose, according to the results of the component analysis. This eventually exposed more fibers for cellulose binding and further improved the sugar release.

## Conclusions

A biomimetic system with a biochemical Fenton reaction and lignocellulose-degrading bacteria was confirmed to be able to pretreatment of RS to enhance enzymatic hydrolysis under mild conditions. The enzymatic hydrolysis results showed that the maximum increase in the digestibility was 56.6% by Fenton + B-8 pretreatment compared with that by Fenton pretreatment alone. Such high digestibility was attributed to the destruction of lignin structure, partial hydrolysis of hemicellulose and partial surface oxidation of cellulose. Multiple detailed physical and chemical characterization steps of RS elaborated the mechanism. This system provided a new insight into the design of more effective strategies for the pretreatment of lignocellulosic biomass.

## Additional files

**Additional file 1: Figure S1.** The effects of pretreatment conditions on the enzymatic hydrolysis of the untreated (dashed line) and pretreated RS. The experimental groups were A: 0.04 M FeCl_3_/0.04 M FeCl_2_/3 M H_2_O_2_; B: 0.03 M FeCl_3_/0.03 M FeCl_2_/2.25 M H_2_O_2_; C: 0.02 M FeCl_3_/0.02 M FeCl_2_/1.5 M H_2_O_2_; D: 0.01 M FeCl_3_/0.01 M FeCl_2_/0.75 M H_2_O_2_.

**Additional file 2: Table S1.** Assignment of lignin and polysaccharide correlation signals in the HSQC spectra shown in Figs. 7, 8.

## Abbreviations

B-8: *Cupriavidus basilensis* B-8
RS: rice straw
2D-NMR: 2D-nuclear magnetic resonance
SEM: scanning electron microscope
AFM: atomic force microscopy
BET: BET specific surface area test method
XRD: X-ray diffraction
CrI: crystallinity index
FTIR: Fourier transform infrared spectroscopy
LCCs: lignin–carbohydrate linkages
*R*_a_: average surface area roughness
*R*_max_: maximum vertical distance between the highest and lowest data points
SSA: specific surface area
PV: pore volume
