# Supplementary material for: A bionic system with Fenton reaction and bacteria as a model for bioprocessing lignocellulosic biomass
Source: Biotechnol Biofuels. 2018 Feb 8;11:31. doi: 10.1186/s13068-018-1035-x (PMC5803899; doi:10.1186/s13068-018-1035-x)
Supplement: Supplementary file 1 — Additional file 1: Figure S1. The effects of pretreatment conditions on the enzymatic hydrolysis of the untreated (dashed line) and pretreated RS. The experimental groups were A: 0.04 M FeCl3/0.04 M FeCl2/3 M H2O2; B: 0.03 M FeCl3/0.03 M FeCl2/2.25 M H2O2; C: 0.02 M FeCl3/0.02 M FeCl2/1.5 M H2O2; D: 0.01 M FeCl3/0.01 M FeCl2/0.75 M H2O2. [file 13068_2018_1035_MOESM1_ESM.docx]

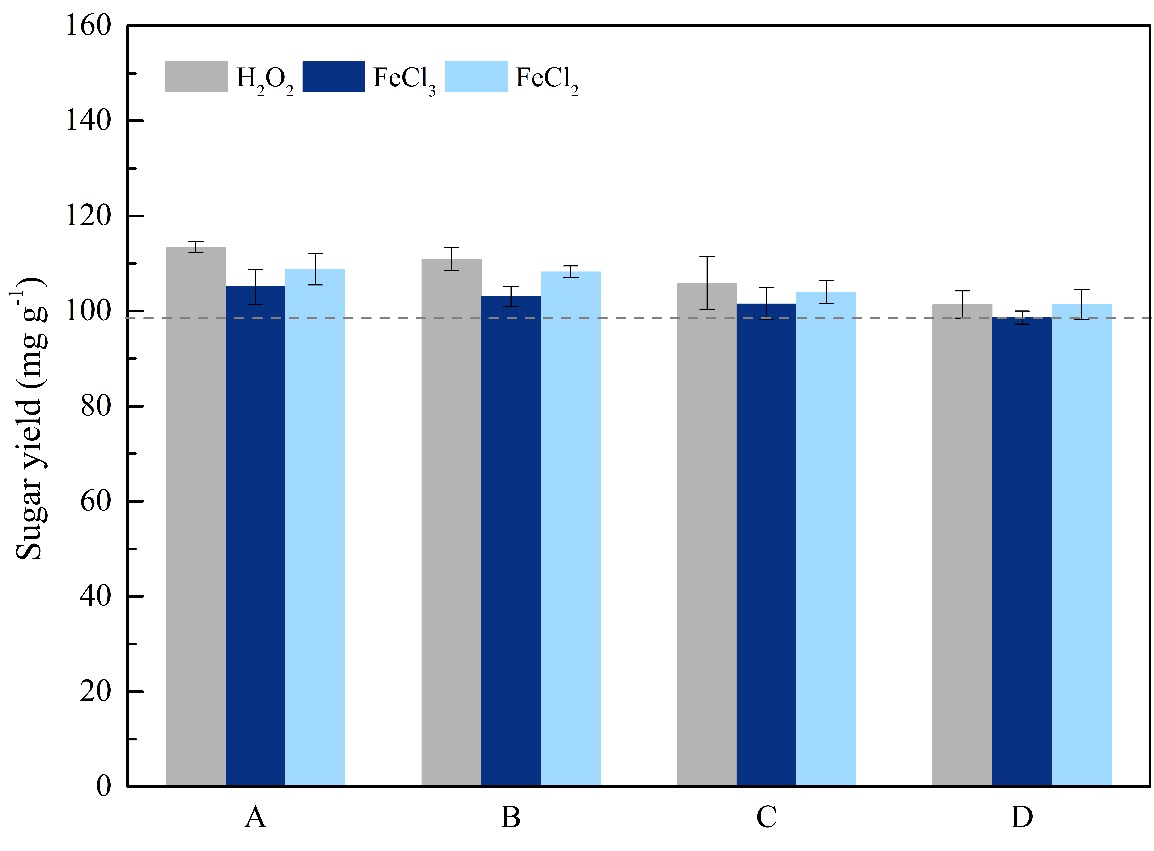


Figure S1 the effects of pretreatment conditions on the enzymatic hydrolysis of the untreated (dashed line) and pretreated RS. The experimental groups were A: 0.04M FeCl_3_/0.04M FeCl_2_/3M H_2_O_2_; B: 0.03M FeCl_3_/0.03M FeCl_2_/2.25M H_2_O_2_; C: 0.02M FeCl_3_/0.02M FeCl_2_/1.5M H_2_O_2_; D: 0.01M FeCl_3_/0.01M FeCl_2_/0.75M H_2_O_2_.

The RS pretreated with only H_2_O_2_ or only Fe(II)/Fe(III) did not obtain effective enzymatic hydrolysis under the same conditions. The oxidability of H_2_O_2_ was greatly low without Fe(II)/Fe(III). In addition, FeCl_3_ solution provided acidic conditions, but sole FeCl_3_ pretreatment under low temperature could not effectively promote the enzymatic hydrolysis of the RS.
