# Supplementary material for: A bionic system with Fenton reaction and bacteria as a model for bioprocessing lignocellulosic biomass
Source: Biotechnol Biofuels. 2018 Feb 8;11:31. doi: 10.1186/s13068-018-1035-x (PMC5803899; doi:10.1186/s13068-018-1035-x)
Supplement: Supplementary file 2 — Additional file 2: Table S1. Assignment of lignin and polysaccharide correlation signals in the HSQC spectra shown in Figs. 7, 8. [file 13068_2018_1035_MOESM2_ESM.docx]

Table S1 Assignment of lignin and polysaccharide correlation signals in the HSQC spectra shown in Fig. 7

| Labels | δ_C_/δ_H_ (ppm) | | | Assignments |
| --- | --- | --- | --- | --- |
| Lignin cross-signals: | | | | |
| OCH_3_ | | 56.2/3.73 | | C–H in methoxyls |
| C_β_ | | 56.3/3.44 | | C_β_-H_β_ in phenylcoumaran (C) |
| B_β_  β | | 54.7/3.10 | | C_β_-H_β_ in *β*-*β*′(resinol) (B) |
| A_γ_ | | 60.2/4.03 | | C_γ_-H_γ_ in *β*-O-4′ substructures (A)  γ  –H  γ  in β–O–4 substructures  γ  –H  γ  in β–O–4 substructures |
| A′_γ_ | | 63.1/3.34 | | C_γ_-H_γ_ in *γ*-acylated *β*-O-4′ (A′) |
| B_γ_ | | 71.2/3.04-3.14 | | C_γ_-H_γ_ in *β*-*β*′ (resinol) (B)  γ  –H  γ  in β-β resinol (B |
| A*_α_* | | 71.7/4.86 | | C_α_-H_α_ in *β*-O-4′ substructures (A) |
| A_β_(G) | | 84.3/4.28 | | C_β_-H_β_ in *β*-O-4′ linked to G (A) |
| B_α_ | | 85.4/4.49 | | C_α_-H_α_ in *β*-*β*′(resinol) (B) |
| A′_β_(G) | | 80.7/4.47 | | C_β_-H_β_ in acylated *β*-O-4′ linked to G (A) |
| A_β_(S) | | 86.4/4.11 | | C_β_-H_β_ in *β*-O-4′ linked to S (A) |
| C_γ_ | | 63.6/3.88 | | C_γ_-H_γ_ in phenylcoumaran (C) |
| I_γ_ | | 60.2/4.03 | | C_γ_-H_γ_ in cinnamyl alcohol end-groups (I) |
| E_α_ | | 79.7/5.55 | | C_α_-H_α_ in *α*, *β*-diaryl ethers (E)  α  –H  α  in α,β-diaryl ethers (E) |
| C_α_ | | 87.7/5.57 | | C_α_-H_α_ in phenylcoumaran (C) |
| S_2,6_ | | 104.4/6.70 | | C_2_-H_2_ and C_6_-H_6_ in syringyl units (S) |
| S′_2,6_ | | 104.7/7.32 | | C_2_-H_2_ and C_6_-H_6_ in oxidized S units (S′) |
| G_2_ | | 111.6/7.28 | | C_2_-H_2_ in guaiacyl units (G) |
| G_5_ | | 115.3/6.72 | | C_5_-H_5_ in guaiacyl units (G) |
| G_6_ | | 119.6/6.77 | | C_6_-H_6_ in guaiacyl units (G) |
| H_2,6_ | | 128.6/7.23 | | C_2,6_-H_2,6_ in H units (H) |
| PCE_3,5_ | | 114.4/6.85 | | C_3,5_-H_3,5_ in *p*-coumarate |
| PCE_2,6_ | | 130.3/7.51 | | C_2,6_-H_2,6_ in *p*-coumarate |
| PCE_7_ | | 144.3/7.49 | | C_7_-H_7_ in *p*-coumarate |
| FA_6_ | | 122.5/7.12 | | C_6_-H_6_ in ferulate |
| Polysaccharide signals: | | | | |
| X_1(R)_ | | 99.4/4.63 | C_1_-H_1_ in *β*-D-xylopyranoside or *β*-D-glucopyranoside (R) | |
| X′_1_ | | 97.1/4.22 | C_1_-H_1_ in 2-*O*-acetyl-*β*-D -xylopyranoside | |
| Gl_1_ | | 102.2/4.25 | C_1_-H_1_ in (1→4) *β*-D -glucopyranoside | |
